# Retraction: Effects of microRNA-19b on airway remodeling, airway inflammation and degree of oxidative stress by targeting TSLP through the Stat3 signaling pathway in a mouse model of asthma

**DOI:** 10.18632/oncotarget.28841

**Published:** 2026-04-17

**Authors:** Ling Ye, Yan Mou, Jian Wang, Mei-Ling Jin

**Affiliations:** ^1^Department of Respiratory Medicine, Affiliated Zhongshan Hospital of Fudan University, Shanghai 200032, P.R. China; ^*^These authors have contributed equally to this work

**This article has been retracted:** Oncotarget has completed its investigation of this paper. Upon investigation, several instances of external image duplication and overlap were found. In particular, Figure 11C, western blot for b-actin, is a duplicate of the GAPDH blot of Figure 4C in an earlier submitted paper [1]. The strong similarity in the paper structure and figures was found with the simultaneously submitted-to-Oncotarget paper from the different institution [2]. This paper was recently retracted. Figure 4 of [2] contains images also found in Figure 4 (HE staining), Figure 5 (Masson staining) and Figure 6 (PAS staining) in the current paper. Figure 4 (HE staining) contains images also found in Figure 1 of [3] and Figure 3A of [4]. Figure 5 (Masson staining) images were found in Figure 4 of [5]. Figure 6 shares PAS stained images with Figure 3B of [4] and Figure 2B of [6]. The authors did not respond to requests to comment on the concerns and provide the raw data. Due to the substantial number of duplications and overlaps in the manuscript, and the absence of original supporting data, the Editorial decision has been made to retract this paper. Oncotarget has reached out to all authors to confirm this retraction, but received no response.

Original article: Oncotarget. 2017; 8:47533–47546. 47533-47546. https://doi.org/10.18632/oncotarget.17258
